# Unveiling antimicrobial activity of microalgae *Chlorella sorokiniana* (UKM2), *Chlorella* sp. (UKM8) and *Scenedesmus* sp. (UKM9)

**DOI:** 10.1016/j.sjbs.2021.09.069

**Published:** 2021-10-02

**Authors:** Abdul Fattah Shaima, Nazlina Haiza Mohd Yasin, Nazlina Ibrahim, Mohd Sobri Takriff, Darvien Gunasekaran, Mahmud Y.Y. Ismaeel

**Affiliations:** a Department of Biological Sciences and Biotechnology, Faculty of Science and Technology, Universiti Kebangsaan Malaysia, 43600, UKM Bangi, Selangor, Malaysia; b Department of Chemical and Process Engineering, Faculty of Engineering and Built Environment, Universiti Kebangsaan Malaysia, 43600 UKM Bangi, Selangor, Malaysia; c Institute of Systems Biology, Universiti Kebangsaan Malaysia, 43600 UKM Bangi, Selangor, Malaysia; d Chemical and Water Desalination Engineering Program, College of Engineering, University of Sharjah, UAE

**Keywords:** Biomass, Extraction, Antibacterial, Cytotoxicity, GC-MS

## Abstract

Microalgae represent promising sources of bioactive compounds for pharmaceutical and industrial applications. The emergence of antibiotic resistant bacteria leads to the need to explore new cost-effective, safe, and potent bioactive compounds from the microalgae. This study aimed to investigate the potential of local microalgae for their antimicrobial properties and bioactive compounds. Three local microalgae namely *Chlorella sorokiniana* (UKM2), *Chlorella* sp. UKM8, and *Scenedesmus* sp. UKM9 biomass methanol extracts (ME) were prepared and tested against Gram-positive and Gram-negative bacteria. *Chlorella* sp. UKM8-ME showed the highest antibacterial activity. UKM8-ME minimum inhibitory concentrations were in the range of 0.312 to 6.25 mg/mL. Cytotoxicity evaluation using MTT assay showed that the microalgae methanolic extracts did not exhibit cytotoxicity against Vero-cells. The UKM8-ME was mainly containing 28 compounds from the Gas Chromatography-Mass Spectrometry (GC–MS) analysis. Major compounds of UKM8-ME included phenol (18.5%), hexadecanoic acid (18.25%), phytol (14.43%), 9,12-octadecadienoic acid (13.69%), and bicyclo[3.1.1]heptane (7.23%), which have been previously described to possess antimicrobial activity. Hence, *Chlorella* sp. (UKM8) methanol extracts showed promising antibacterial activity. More comprehensive studies are required to purify these antimicrobial compounds and develop our understanding on their mechanism in UKM8-ME to unleash their specific potential.

## 1 Introduction

Malaysia has great microalgae diversity in a variety of habitats (Phang et al., 2015, Shaima et al., 2016). Microalgae can survive in a robust environment due to their adaptive survival strategy that may include the production of novel and distinctive biologically compounds (Caldwell, 2009, Landsberg, 2002). These active molecules are useful in the applications of food, medicinal, nutraceutical, and cosmetic industries (Guedes et al., 2013, Mimouni et al., 2012). Microalgae become the potential valuable source for new active compounds as they are easy to cultivate at short generation time, environmental friendly, and renewable. To date, no extensive reports have been investigated on microalgae for drug discovery (Lauritano et al., 2016) that makes them of great choice in drug development.

Various valuable active compounds have been determined from microalgae such as carotenoids, phycocyanin, phenolics, amino acids, polyunsaturated fatty acids, sulphated polysaccharides, pigments, lipids phlorotannins, polysaccharides, peptides, terpenes, polyacetylenes, sterols, indole alkaloids, aromatic organic acids, shikimic acid, polyketides, hydroquinones, alcohols, aldehydes, ketones, halogenated furanones, alkanes, and alkenes (Marrez et al., 2019, Shannon and Abu-Ghannam, 2016, Vikneshan et al., 2020). These compounds were related to a range of pharmacological activities including, antimicrobial, antioxidant, antiviral, antitumor, anti-inflammatory, and anti-allergy effects (Lauritano et al., 2016, Patra et al., 2009, Shannon and Abu-Ghannam, 2016).

The increasing number of reported cases on antibiotics resistance is of global concern (Prestinaci et al., 2015, Yap et al., 2019). To this problem, the discovery of new antibiotics or compounds with antibacterial properties is necessary and in urgent need (Chanda and Rakholiya, 2011). Antibacterial activity from microalgae extracts have been studied by various researchers (Chanda and Rakholiya, 2011, de Morais et al., 2015, Kellam and Walker, 1989). The green microalgae from genus *Chlorella* spp. and *Scenedesmus* spp. are valuable sources of a wide range of bioactive compounds especially with antimicrobial activity (Wolfe et al., 2002). Several studies reported the antimicrobial activity of both species against different species of pathogenic bacteria (Jafari et al., 2018, Zielinski et al., 2020).

This study aimed to explore the antibacterial activity of three methanol extracts from the biomass of local microalgae: *Chlorella* sp. UKM2, *Chlorella* sp. UKM8 and *Scenedesmus* sp. UKM9. These microalgae isolates have been reported to have potential in phycoremediation (Ding et al., 2020, Hariz et al., 2019, Hazman et al., 2018)but their bioactive potentials are yet to be discovered. This study determined the antibacterial and cytotoxicity activity of the microalgae extracts. The bioactive compounds found in the extract with antibacterial properties were identified.

## 2 Methods

### 2.1 Microalgae cultivation and biomass extraction

Bold Basal Media (BBM) was used as the cultivation media for the local microalgae isolates, *Chlorella* sp*.* UKM2, *Chlorella.* sp. UKM8, and *Scenedesmus* sp. UKM9. Cultures were prepared independently in sterile conditions with 30% (v/v) of inoculum size. The BBM medium was composed of the following components (g/L): K_2_HPO_4_ (75), MgSO_4_·7H_2_O (75), CaCl_2_·2H_2_O (25), H_3_BO_3_ (11.4), NaCl (25), EDTA.Na_2_ (50), NaNO_3_ (2 5 0), KH_2_PO_4_ (173.8), FeSO_4_·7H_2_O (4.98), ZnSO_4_·7H_2_O (8.82), H_2_SO_4_ (1), MnCl_2_·4H_2_O (1.44), MoO_3_ (0.71), CuSO_4_·5H_2_O (1.572), and Co (NO_3_)_2_·6H_2_O (0.49). The incubation temperature was maintained at 25 ± 2 °C with a sufficient supply of light and air. Culture flasks were shaken twice a day and placed in the growth chamber. Microalgae growth was evaluated during a cultivation period of 15 days using biomass dry cell weight (DCW) measurement. In every 24 h, the cultures were filtered using pre-heated GFC-Filter (Whatman Filter Paper) and dried at 105 °C in an oven overnight until a consistent weight value was achieved. The value difference of GFC-Filter weight before and after filtration equals the value of biomass produced as shown in Eq. (1):

(1) y=Xf-XoV

where y is the biomass produced (mg/L), *X*o is the weight of GFC filter paper before filtration with microalgae sample, and *X*f is after filtration. V is the volume of microalgae solution used for the filtration process.

The growth kinetics of the microalgae were determined by logistic equation based on the dry cell weight analysis results. The maximum specific growth rate, μmax, was obtained from the logistic model as shown in Eq. (2):

(2) $$X=\frac{\mathrm{XoXmax}e^{\mu maxt}}{(\left(Xmax-Xo\right)+Xoe^{\mu maxt}}$$

where X is microalgae concentration in the medium, μmax is the maximum specific growth rate, Xo is the initial concentration, and Xmax is the maximum microalgae concentration.

For biomass extraction, cells were harvested when the culture O.D reach 0.9 ± 0.1 (approximately 10 days) by centrifugation at 2862 *xg* for 10 min. One g of biomass was immersed in 100 mL methanol overnight at room temperature. The extract was filtered through Whatman No. 1 filter paper to remove all non-extractable matters including cellular material. The filtrate was concentrated and dried in a rotary evaporator. Concentrated extracts were stored in vials and weighed. The extracts were left to dry in the fume chamber until they achieved a constant weight. Dry weight percentage was recorded and stored at 4 °C for future used.

### 2.2 Bacterial strain

*Pseudomonas aeruginosa* ATCC 10145, the three clinical *P. aeruginosa* strains, *Staphylococcus epidermidis* ATCC 12228, *S. aureus* ATCC 25923, *Escherichia coli* ATCC 10536, Methicillin-resistant *S. aureus* ATCC 43300, *Shigella sonnei* ATCC 2993, *Bacillus subtilis* UKMCC1002, *B. subtilis* ATCC 11774, *Serratia marcescens* UKMCC0014, *B. thuringiensis* ATCC10792, three clinical *B. cereus* strains, *Enterobacter faecalis* ATCC 14506, and *Klebsiella pneuomoniae* ATCC BAA1144 were obtained from the stock culture in the Microbiology Laboratory, Department of Biological Sciences and Biotechnology, Faculty of Science and Technology, Universiti Kebangsaan Malaysia. All the bacterial strains were sub-cultured in Brain Heart Infusion (BHI) broth and incubated on the rotating shaker at 37 °C, 200 rpm for 24 h. Each culture was then streaked on a nutrient agar medium.

### 2.3 Antibacterial assay

The antibacterial evaluation was performed using the well and agar disc diffusion methods according to Patra et al., 2009, Bauer et al., 1966, respectively. About 200 mg of the tested microalgae-ME was dissolved in 5% (v/v) Tween 20 and 10% (v/v) DMSO. For the disc diffusion method, inoculum suspension of bacteria with 0.5 McFarland standards was streaked on Mueller-Hinton Agar surface and allowed to dry. Sterile Whatman No. 1 filter paper with a diameter of 6 mm was impregnated with the tested microalgae-ME, air-dried, and placed on the bacterial lawn. For the well diffusion method, the agar plate surface was inoculated with a lawn of bacteria. A hole with a diameter of 6 mm was punched aseptically with a sterile cork borer and the extract or antibiotic at desired concentration was introduced into the well. The culture plates were kept for pre-diffusion for 1-h prior to incubation at 37 °C for 24 h. Gentamicin (10 µg) and vancomycin (30 µg) were used as the antibiotic controls. The negative control was sterile 5% (v/v) Tween 20 and 10% (v/v) DMSO. The diameter of the inhibition zone was measured in millimetre (mm). The experiment was performed in triplicates, and the mean of the inhibition zone was calculated.

### 2.4 Determination of minimum inhibitory concentration (MIC)

The antimicrobial activity of UKM8-ME was further tested using the minimum inhibitory concentration (MIC). The crude extract was prepared in 5% (v/v) Tween 20 and 10% (v/v) DMSO. The stock was serially diluted two-fold in Mueller Hinton broth (MHB) in 96 well microtitre plates to a final volume of 100 μL. Test bacteria suspensions were prepared to a density to 0.5 McFarland standards. The microalgae extracts or antibiotic control were added to a final volume of 200 μL/well. Wells containing only sterile MHB was used as a negative control and gentamicin serves as a positive control. After 24 h of incubation at 37 °C, 5 μL of 3-(4, 5-dimethyl-2-thiazolyl)-2,5 diphenyltetrazolium bromide (MTT) (Sigma Chemical Co.) (5 mg/mL) was added to each well. Plates were further incubated at 37 °C for 4 h. MTT indicates bacterial growth when yellow tetrazolium bromide was reduced to violet formazan. All assays were performed in triplicates.

### 2.5 Cytotoxicity assay

The cytotoxicity of microalgae-ME was evaluated in Vero cells (African green monkey kidney cells) according to Fayyad et al. (2014). Vero cells were maintained in Dulbecco’s Modified Eagle’s Medium (DMEM) supplemented with 5% (v/v) fetal bovine serum (FBS). Confluent cells were grown in 96-well microtitre plates at a density of 2 × 10^5^ cells/well. The plates were incubated for 24 h at 37 °C in a humidified 5% (v/v) CO_2_ atmosphere. The medium was removed and the cells were retained in the plates. The cells were exposed with 100 µL of dilutions of the microalgae ME at the concentration in the range of 10 to 0.0195 mg/mL prepared in 2% (v/v) dimethyl sulfoxide (DMSO) and cell culture media in each well. The negative control was prepared using the culture medium without cells. Plates were further incubated for 48 h. The medium was removed, cells were washed with PBS and treated with 30 μL of MTT solution. Cells were then incubated for 3 h. The MTT solution was removed and 100 μL of DMSO was added to each well to solubilize the formazan crystals. Plates were slightly shaken until formazan crystals completely dissolved. The absorbance for each well was determined at 540 nm in a multiwall spectrophotometer (Bio-Rad 680, USA). The cytotoxic concentration that kills 50% of the cell population (CC_50_) was determined using Graph Pad Prism 8.

### 2.6 GC–MS analysis

The UKM8-ME (500 µg/mL) was sent for compound analysis to Makmal Pencirian Struktur Molekul (MPSM), Center for Research and Instrumentation Management (CRIM), Universiti Kebangsaan Malaysia. The sample was analysed on Agilent 7890A gas chromatograph (GC) (USA) directly coupled to the mass spectrophotometer system (MS) of Agilent 5975C inert MSD with a triple-axis detector.

## 3 Results

### 3.1 Microalgae growth and methanol extract yield

The growth rate of the three microalgae species was assessed by their biomass. UKM2, UKM8, and UKM9 exhibited a specific growth rate (μmax) of 0.3877, 0.4476, and 0.4465 day^−1^, respectively. The biomass and the methanol extract yield at the late exponential phase are presented in Table 1.

**Table 1:** Biomass and the yield of methanol crude extract of UKM2, UKM8 and UKM9 isolates.

Microalgae	Biomass total solid (mg/L)	Methanol extract yield (mg/g)
UKM2	950	158
UKM8	745	190
UKM9	850	251

### 3.2 Antimicrobial activity

**Fig. 1:**
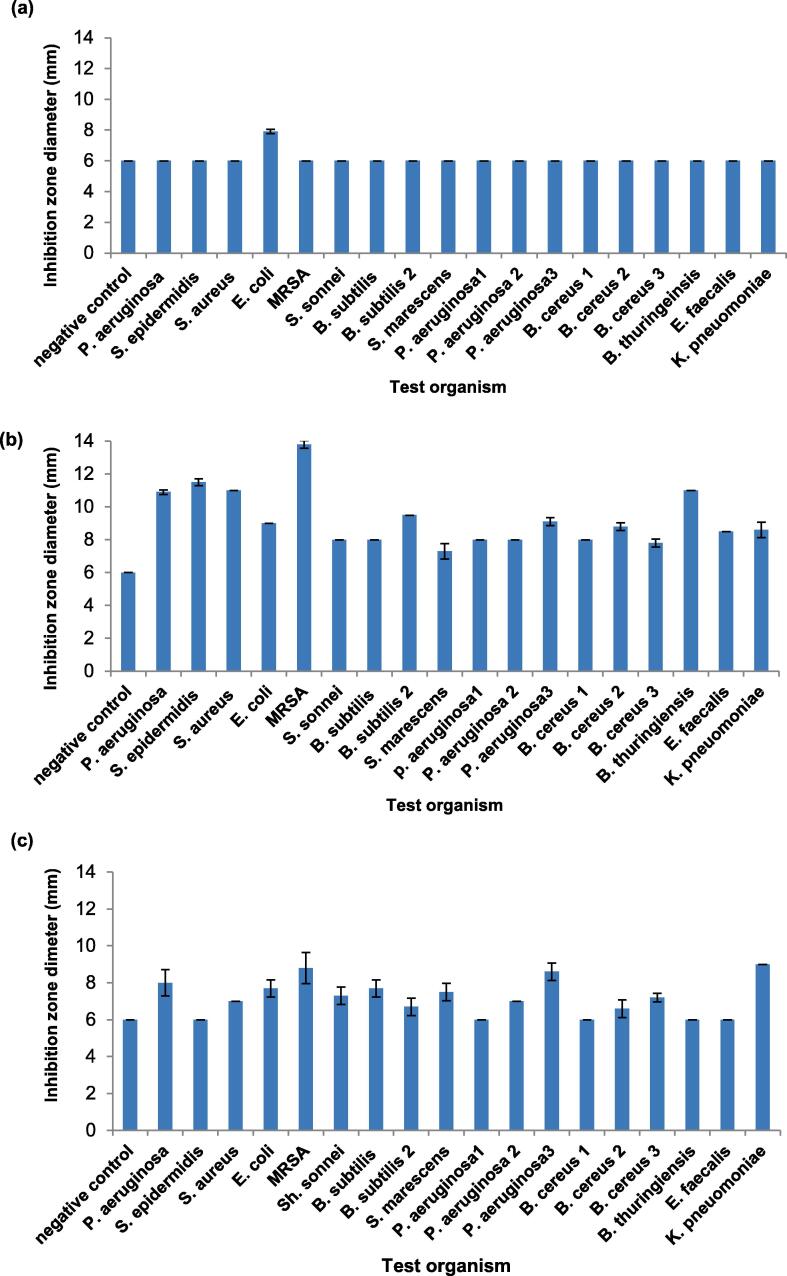
Antibacterial activity evaluation of methanolic extract (ME) from microalgae (a) UKM2 (b) UKM8 (c) UKM9 using disc diffusion method. Results were expressed by the diameter size of the inhibition zone (mm). Diameter of disc was 6 mm. The negative control was sterile 5% (v/v) Tween 20 and 10% (v/v) DMSO.

The methanol extracts of the three microalgae species were tested in disc diffusion assay and the diameter of inhibition zones are shown in Fig. 1. UKM8-ME showed excellent activity against MRSA (13.8 mm), *S. epidermidis* (11.3 mm), *S. aureus* (11 mm), *B. thuringiensis* (11 mm), *P. aeruginosa* (10.9 mm), *E. coli* (9 mm) and two clinical strain of *B. subtilis* (9.1 and 9.5 mm). UKM2-ME displayed the lowest activity by inhibiting *E. coli* only. UKM9-ME has limited antibacterial activity towards 13 isolates of *P. aeruginosa* ATCC 10145, *S. aureus, E. coli*, MRSA, *Sh. sonnei*, *B. subtilis,* the clinical strain *B. subtilis* (2), S. *marcescens,* two clinical strains of *P. aeruginosa* (2 and 3), *K. pneuomoniae, and clinical strain of B. cereus* (2 and 3).

In the well diffusion method, both UKM2-ME and UKM9-ME did not exhibit antibacterial activity (data not shown). However, UKM8-ME was active against one isolate (*P. aeruginosa* ATCC 10145) with the 7.5 ± 0.4 mm of inhibition zone. The results indicate that the disc-diffusion assay offers better inhibition ability compared to the well diffusion method. The disc-diffusion assay was reported as the most prominent method to check for antimicrobial activity for enormous bacteria compared to the well diffusion method, with the advantage as a convenient and cost-effective method (Balouiri et al., 2016). The disc-diffusion assay is more sensitive towards a wide range of fastidious bacteria. Table 2 shows that UKM8-ME achieved the largest inhibition zone against MRSA with 13.8 mm. The data can be almost correlated to the reference drug (Vancomycin (30 µg) and Gentamycin (10 µg)) with the inhibition zone in the range of 14–21 mm.

**Table 2:** Inhibition zone (mean diameter of inhibition in mm) and minimum inhibition concentration (MIC) of UKM8- ME against tested bacteria.

Isolates	Inhibition zone (mm)			MIC (mg/mL)
	UKM8-ME	Rd^a^	Rd^b^	
*P. aeruginosa*	10.9	nt	19	5
*S. epidermidis*	11.5	17	20	2.5
*S. aureus*	11	17	21	1.25
*E. coli*	9	nt	17	2.5
*MRSA*	13.8	18	21	0.39
*S. sonnei*	8	nt	20	5
*B. subtilis*	8	16	20	5
*B. subtilis* 2	9.5	16	19	2.5
*S. marcescens*	7.3	nt	20	6.25
*P. aeruginosa1*	8	nt	19	5
*P. aeruginosa 2*	8	nt	20	5
*P. aeruginosa 3*	9.1	nt	20	3.125
*B. cereus 1*	8	14	19	5
*B. cereus 2*	8.8	16	18	6.25
*B. cereus 3*	7.8	16	18	5
*B. thuringiensis*	11	15	19	1.25
*E. faecalis*	8.5	18	16	2.5
*K. pneuomoniae*	*8.6*	nt	14	*5*

nt, not tested; Rd^a^ , vancomycin 30 µg, Rd^b^, gentamicin 10 µg.

Rd^a^ was not tested (nt) to Gram-negative bacteria due to the unsuitability.

### 3.3 Minimum inhibitory concentration (MIC)

Minimum inhibitory concentration (MIC) was conducted in this study to determine quantitative antibacterial values. MIC is the lowest concentration of antibiotic or extracts that completely inhibits the visible growth of the test organisms. Only UKM8-ME was evaluated due to its performance in the antibacterial screening compared to other microalgae as indicated in Fig. 1. Table 2 shows the comparison of inhibition zone from disc-diffusion method and MIC assay using the methanolic extract of UKM8. Antibacterial activity of UKM8-ME was shown at all selected MIC concentrations using the microdilution method with the range between 0.312 and 6.25 mg/mL.

### 3.4 Cytotoxicity assay

**Fig. 2:**
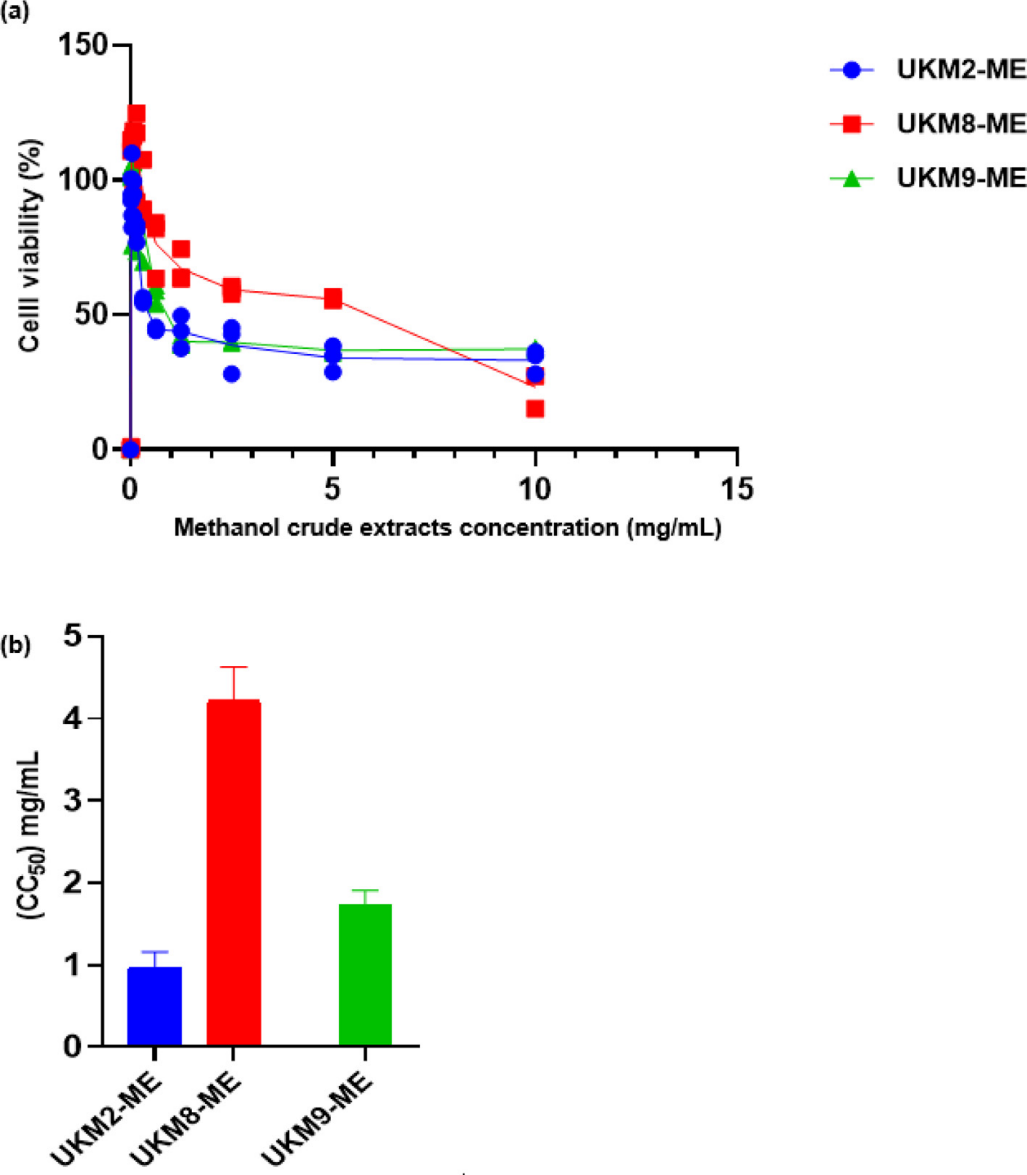
Cytotoxicity evaluation of methanolic extract of UKM2, UKM8 and UKM9 using MTT assay against Vero cell line. (a) The Vero cells viability at different methanol crude extracts concentration and (b) CC_50_ values of UKM2, UKM8 and UKM9.

Vero cells survival exposed to UKM2-ME, UKM8-ME, and UKM9-ME at different concentrations is shown in Fig. 2a. CC_50_ values obtained from UKM2, UKM8, and UKM9 were 0.971, 4.21, and 1.734 mg/mL, respectively (Fig. 2b).

### 3.5 Compound identification by GC–MS.

**Fig. 3:**
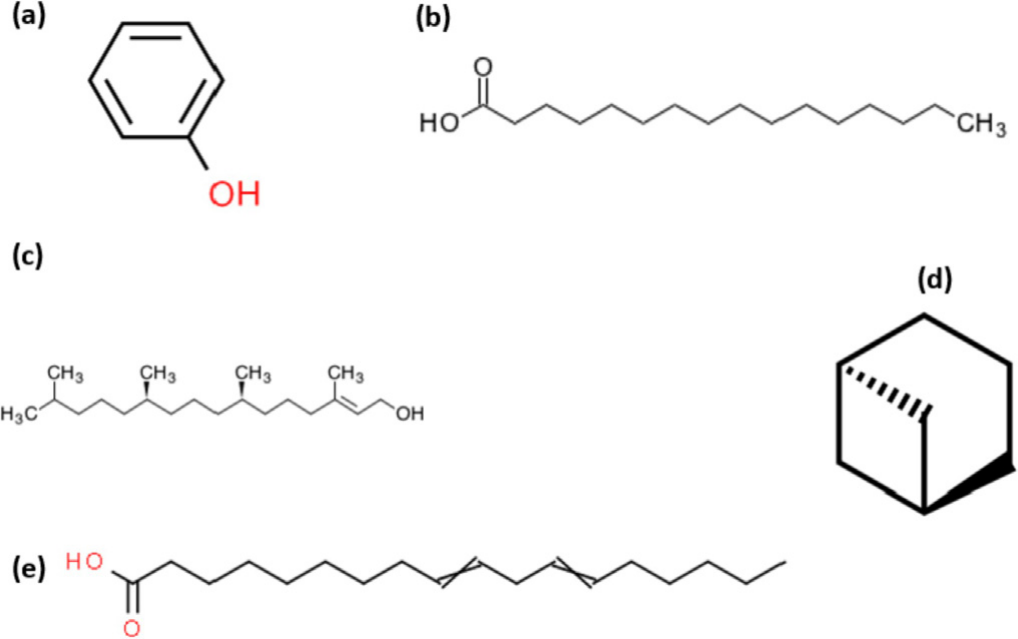
Structure for the abundant compounds found in GC–MS peaks. (a) Phenol, (b) Hexadecanoic acid, (c) phytol, (d) Bicyclo[3.1.1]heptane (E) 9,12- Octadecadienoic acid. Chemical structure of phenol, hexadecanoic acid and phytol retrieved from KEGG online database, while bicyclo[3.1.1]heptane and 9,12- Octadecadienoic acid from Chemspider online data base.

Further identification of the active compounds in UKM8-ME was carried out based on their profound antibacterial activities. The identification was performed using mass spectrometry (MS) based on the comparison of mass spectra and retention index (RI). GC–MS is a useful technique in natural product research for major and minor compound identification due to its sensitivity and ability to provide accurate mass measurements (Owen et al., 2019). Table 4 shows the compounds detected in UKM8-ME with peak area percentages ranging from 0.2 to 14.43%. Diversity of the classes composed of fatty acids, alkanes, alkenes, and alcohols. The chemical composition profile of UKM8 has not been reported before. Therefore, our results can be evaluated as the first report about the composition of the methanolic extract of this native species. Phenol, hexadecanoic acid, phytol, 9,12-octadecadienoic acid, and bicyclo[3.1.1]heptane were the major contributors. Chemical structures were retrieved from KEGG and Chemspider online database as illustrated in Fig. 3 .

**Table 3:** Inhibition zone (mean diameter of inhibition in mm) of UKM8-ME and UKM9-ME compared to literature study using *Chlorella* sp, and *Scenedesmus* sp.

Bacterial isolates	*Chlorella* sp. UKM8-ME	*Scenedesmus* sp. UKM9-ME	*C. stigmatophora*	*S. obliquus*	*C. vulgaris*	*C. vulgaris*	*Chlorella* sp.
*P. aeruginosa*	8–10.9	7–8	-	N	–	–	12
*S. epidermidis*	11.5	N	–	–	–	–	–
*S. aureus*	11	7	6.5	9.7	9	17	15
*E. coli*	9	7.7	–	9.7	–	–	17
*MRSA*	13.8	8.8	–	–	–	–	–
*Sh. sonnei*	8	7.3	–	–	–	–	–
*B. subtilis*	8–9.5	7–8	N	–	–	17.5	19
*S. marescens*	7.3	7.5	–	–	–	–	14
*B. cereus*	7 to 9	7	–	9	–	–	22
*B. thuringiensis*	11	N	–	–	–	–	–
*E. faecalis*	8.5	N	–	–	N	–	16
*K. pneuomoniae*	8.6	9	–	N	N	14.5	–
References	This study	This study	(Kellam and Walker 1989)	(Marrez et al., 2019)	(Thamilvanan et al., 2016)	(Salem et al., 2014)	(Santhosh et al., 2019)

N,negative; -, not tested.

**Table 4:** Compounds obtained by GC–MS profiling UKM8-ME, their classification, peak area percentage, molecular name (MW), molecular formula and activity.

Classification	Compound name	Peak area %	MW (g/mol)	Molecular formula	Activity	References
**Fatty acids**	7,10,13-hexadecatrienoic acid	2.81	250.38	C_16_H_26_O_2_	Antimicrobial	(Cifuentes et al., 2006)
	7,10-Hexadecadienoic acid	3.73	252.39	C_16_H_28_O_2_	Antioxidant, antimicrobial, anti-inflammatory	(Gheda and Ismail, 2020)
	8,11-Octadecadienoic acid	0.85	280.4	C_18_H_32_O_2_	Antimicrobial	(Hassan et al., 2016)
	9,12,15-Octadecatrienoic acid	0.71	278.4	C_18_H_30_O_2_	Antimicrobial	(Plaza et al., 2010)
	9,12-Octadecadienoic acid	13.97	280.4	C_18_H_32_O_2_	Antimicrobial, antioxidant	(Farooqui et al., 2019)
	Cis-13-Octadecenoic acid	1.01	282.5	C_18_H_34_O_2_	Antimicrobial	(Abdelrheem et al., 2020)
	Heptadecanoic acid	1.29	270.5	C_17_H_34_O_2_	Antimicrobial, antifungal	(de Felício et al., 2010)
	Hexadecanoic acid	18.25	256.42	C_16_H_32_O_2_	Anticarcinogenic, anti-inflammatory and antimicrobial activity	(Flickinger and Huth, 2004)
**Alkane**	1-bromodocosane	2.47	389.5	C_22_H_45_Br	Antimicrobial	(Nand et al., 2011)
	Bicyclo[3.1.1]heptane	7.23	175.07	C_7_H_12_	Antimicrobial	(Olasehinde et al., 2019)
	Cis-8-methyl-*exo*-tricyclo[5.2.1.0(2.6)]decane	1.89	150.26	C_11_H_18_	Antimicrobial, antioxidant	(Jodallah and saleem Ali-shtayeh, 2013)
	Cyclotetradecane	1.30	196.37	C_14_H_28_	Antimicrobial	(Adhoni et al., 2016)
	Docosane	1.52	310.6	C_22_H_46_	Antimicrobial	(Karabay-Yavasoglu et al., 2007)
	Heptacosane	1.74	380.7	C_27_H_56_	Antimicrobial	(Silva et al., 2020)
	Tetrapentacontane	1.77	759.4	C_54_H_110_	Antimicrobial	(Ramasamy, 2014)
	Heptadecane	0.83	240.5	C_17_H_36_	Antimicrobial	(Ozdemir et al., 2004)
	Eicosane	1.27	282.5	C_20_H_42_	Antimicrobial, antioxidant	(Demirel et al., 2009)
	Tetracosane	0.38	338.7	C_24_H_50_	Antimicrobial	(Karabay-Yavasoglu et al., 2007)
	Tetratriacontane	0.63	478.9	C_34_H_70_	Antimicrobial	(Karabay-Yavasoglu et al., 2007)
	Tricosane	0.64	324.6	C_23_H_48_	Antimicrobial	(Ozdemir et al., 2004)
	Tridecane	1.37	184.36	C_13_H_28_	Antimicrobial	(Karabay-Yavasoglu et al., 2007)
	7-Tetradecyne	0.30	194.36	C_14_H_26_	Antimicrobial	(Bhaigybati et al., 2020)
**Alkene**	1-nonadecene	1.50	266.5	C_19_H_38_	Antimicrobial	(Hussein et al., 2020)
	Cetene	1.06	224.42	C_16_H_32_	Antimicrobial, antioxidant	(Kamat et al., 2020)
	Z-12-Pentacosene	0.76	350.7	C_25_H_50_	Antimicrobial, antioxidant	(Lv et al., 2011)
	9-Hexacosene	0.79	364.7	C_26_H_52_	Antimicrobial	(Mohamed and Saber, 2019)
**Phenol and phytol**	Phytol	14.44	296.5	C_20_H_40_O	Antimicrobial	(Sawant and Mane, 2018)(Kumar, 2011)
	Phenol	18.50	94.11	C_6_H_5_OH	Antimicrobial	(Sawant and Mane, 2018) ,(Bajpai, 2016).

## 4 Discussion

### 4.1 Microalgae growth performance and extracts yield

UKM2, UKM8 and UKM9 exhibited diversity in growth performance (μmax). UKM8 presented relatively higher specific growth rate among the three isolates. Growth rate of microalgae diversity can be explained by microalgae adaptability hence high growth rate reflecting high adaptability of microalgae species toward growth conditions. The diversity in growth performances is due to the difference in microalgae species (Japar et al., 2021).

UKM2 showed the highest biomass with no significant difference between ME yields, indicated that methanol extract yield was not correlated to the total biomass. Cell wall structure and chemical content in microalga especially from different classes are known to vary in their cell wall structure and chemical composition (Hoek et al., 1995). They may contain cellulose, pectin, and other compounds in different arrangements and proportions. Biomass production is very related to the culture condition, which can be optimized according to the specific microalgae strain to obtain the optimum yield (Mudimu et al., 2014).

### 4.2 Antimicrobial activity

Methanol extraction is an established and well-reported method to isolate active antimicrobial components from microalgae (Patil and Kaliwal, 2019, Zea-Obando et al., 2018). Table 3 shows the obtained ME from UKM8 and UKM9 were effective against a broader spectrum of Gram-positive and Gram-negative bacteria, which could be considered as promising antibacterial agents. The antibacterial activity of *Scenedesmus* sp. UKM9 against MRSA, *Sh. sonnei,* and *S. marcescens as well as* the antibacterial activity of *Chlorella* sp. UKM8 against *Sh. sonnei* has never been reported in the literature, indicating the interest of this extract to be further investigated.

The antibacterial activity of UKM8-ME was found to be more excellent against Gram-positive bacteria, including MRSA, *S. epidermidis*, *S. aureus*, and *B. thuringiensis* with inhibition zone in the range of 11–13.8 mm*.* The highest inhibition zone (13.8 mm) is almost comparable to the positive control (Vancomycin and Gentamycin) with the minimum inhibition zone of 14 mm (Table 2). Gram-positive bacteria also proved to be the most susceptible to algal extracts in several studies (Kellam and Walker, 1989, Reichelt and Borowitzka, 1984). This observation depends on the compounds extracted from algae and other biological sources have been shown to be more effective against Gram-positive bacteria than Gram-negative. For example, Kamei and Isnansetyo reported that the bacteriolytic activity of phloroglucinol compouds isolated from *Pseudomonas* sp*.* against *Vibrio parahaemolyticus* required a greater (MIC), compared to the Gram-positive MRSA (Kamei and Isnansetyo, 2003) . This is mainly due to the different cell wall structures between the two bacteria groups. Gram-negative bacteria have an additional outer membrane that acts as a protector against toxic material such as antibiotics. This layer is composed of glycerol phospholipids and glycolipid lipopolysaccharides. It also has the capability of interpreting bacterial signals from compounds that can destroy the cell. Destruction to the outer membrane can also be detected and repaired.

Additionally, Gram-negative bacteria possess porin channels that can prevent the entry of toxic chemicals and antibiotics. These channels can also eject antibiotics that make the Gram-negative bacteria becomes much more challenging to treat than Gram-positive bacteria (Makridis et al., 2006). Therefore, the outer membrane plays an essential role in protecting bacteria against harmful agents. Gram-positive bacteria do not have an outer membrane protein, therefore, UKM8-ME was effective for these bacteria groups. The cell wall contains a thick layer of peptidoglycan with no effective permeability barrier making the cell wall more susceptible to antibiotics.

The efficiency of antibacterial activity of UKM8-ME and UKM9-ME were compared to the literature study, which used bioactive compounds from green microalgae, *Chlorella* sp. and *Scenedesmus* sp. against several pathogens as shown in Table 3. The values showed that UKM8-ME and UKM9-ME extracts inhibits bacteria with diameter of inhibition zone (ranges from 7 to 13.8 mm) are comparable to that reported in the literature (ranged from 6.5 to 22 mm). Extract from *Chlorella* sp. was very effective as antibacterial agent against *S. aureus, E. coli, B. subtilis and B. cereus,* which have been noted previously by (Kellam and Walker, 1989, Salem et al., 2014, Santhosh et al., 2019, Thamilvanan et al., 2016). Among genus *Scenedesmus*, the inhibition effect of ME from *S. obliquus* was found active against *E. coli*, *B. cereus*, and *S. aureus* with an inhibition zone between 9 and 9.7 mm (Marrez et al., 2019). Those findings are relatively comparable to our antibacterial observations with an inhibition zone up to 13.8 mm.

Antibacterial activity of UKM8-ME has MIC values ranging from 0.312 to 6.25 mg/mL. Among all test organisms, MRSA, *S. aureus, B. thuringiensis*, *S. epidermidis, E. coli, B. subtilis* 2, *and E. faecalis* showed the lowest MIC values of UKM8-ME (Table 2). Jafari et al., (2018) studied the antimicrobial activity of *C. vulgaris* and *Dunaliella salina* ME against *S. mutans* with MIC values of 5 and 6.5 mg/mL, respectively. Meanwhile, another study reported the antimicrobial activity of *Chlorella* and other methanolic extracts of microalgae against *E. coli*, *S. aureus*, and *P. aeruginosa* with the MIC values in the range of 2.6 to 5 mg/mL (Maadane et al., 2015). The range of MIC values reported in the previous reports was consistent with the range found in this study. MIC and disc diffusion results of UKM8-ME were relatively compatible and they showed antibacterial activities against all selected bacteria with different susceptibilities.

### 4.3 UKM2, UKM8 and UKM9 extracts cytotoxicity

Cytotoxicity of UKM2, UKM8 and UKM9 ME was tested on Vero cells using MTT test. The main purpose of MTT is to measure the relative survival of cells through the measurement of high throughput performed in a 96-well plate without the need for complex cell calculations (Meerloo et al., 2011). CC_50_ values of UKM2, UKM8 and UKM9 ME were 0.971, 4.21 and 1.734 mg/ml, respectively. According to Malebo et al., (2009), CC_50_ values above 30 μg/mL for the extract are considered as non-toxic. Hence, all three microalgae extracts in this study were non-cytotoxic.

Methanolic extract of other green microalgae has also previously been reported as non-cytotoxic (Maadane et al., 2015). Toxicity of an extract may be attributed to its metabolites content (Sit et al., 2018, Malebo et al., 2009). The low toxicity of an extract indicates that its active compounds have been thoroughly investigated and subsequently used as promising antibacterial agent (Coronado-reyes et al., 2020). This is a significant feature of an extract as the source of biologically active compounds for pharmacological purposes. Therefore, the microalgae extracts should be further investigated for development in antibacterial application and other biological activities.

### 4.4 Compound identification by GC–MS

The main compounds contributors that identified in UKM8-ME were phenol, hexadecanoic acid, phytol, 9,12-octadecadienoic acid, and bicyclo[3.1.1]heptane. Phenol can be found widely in nature. The structure of the phenol is illustrated in Fig. 3a. The chemical composition of phenol differs from one aromatic set to very complex polymerized molecules. Phenol is subjected to a range of MIC levels ranging from 0.1 to 10 mg/mL for different Gram-negative and Gram-positive bacterial pathogens (Bajpai, 2016). Therefore, there is no doubt about the effectiveness of this compound against the pathogens used in this study. Phenol has also been detected in *Chlorella* sp. (Sawant and Mane, 2018) and other microalgae with proven antimicrobial potential (Bajpai, 2016).

Fatty acids (FAs) are long, unbranched carbon chain carboxylic acids, in which some chains can include double bonds. The number of carbon chains of the biological system varies from 10 to 28. The structure of FAs composed of the carboxylic (–COOH) group at one side of the carbon chain, and the methylic (–CH3) group at the opposite end. FAs are known to be the long chain of 16 carbon atoms. Unsaturated FAs have one or more double bonds C = C on the carbon chain, while saturated FAs have single bonds C-C linked with carbon atoms. The structure of FAs has been found to influence their ability to lyse microbes (Desbois and Smith, 2010). Studies have shown that FAs have been selectively inhibiting or interrupting various microbial pathogens (Kumar et al., 2020). Microalgae are good choices for evaluating the antibacterial potential of the FAs mixture, since the individual microalgae have their specific fatty acids composition, depending on the taxonomic and growth conditions (Saritha et al., 2017).

In the present study, hexadecanoic acid (Fig. 3b) and 9,12-Octadecadienoic acid (Fig. 3e) were the major FA compounds in UKM8-ME. Hexadecanoic acid is a saturated fatty acid (16:0). Meanwhile, 9,12-Octadecadienoic acid is a polyunsaturated fatty acid (18:2). These FAs have been reported in *Chlorella emersonii,* which demonstrated antimicrobial activity (Elshobary et al., 2020, Sawant and Mane, 2018). Moreover, several researchers have correlated the effect of FAs with different pathological situations such as positive effects against cardiovascular diseases, anti-carcinogenic, anti-inflammatory, and anti-microbial activity (Flickinger and Huth, 2004). Therefore, *Chlorella* possesses useful metabolites with major health benefits for humans.

Phytol is a long-chain, unsaturated acyclic alcohol diterpene member (Fig. 3c). This compound and some of its derivatives, such as plant acid (PA) have various biological effects (Islam et al., 2018). Phytol was one of the main compounds in UKM8-ME. This result was in accordance with (Sawant and Mane, 2018), who stated the presence of phytol as a major compound in *C. emersonii* with antimicrobial activity. Phytol has also been reported for its antimicrobial activity in certain microalgae (Kumar, 2011). In addition to the antimicrobial activity, this compound has also been described for its antioxidant, anti-inflammatory, anticancer, diuretic, anti-malarial, and anti-mycobacterial (Plaza et al., 2012, Sawant and Mane, 2018).

Bicyclo [3.1.1] heptane is categorised in the alkane group (Fig. 3d), which was found abundant in UKM8-ME (Table 4). This similar compound was previously found in *C. sorokiniana* with antimicrobial activity (Olasehinde et al., 2019). The promising antimicrobial property of these bioactive compounds requires further multi-pronged studies as a novel therapeutic agent to treat ailments of the drug-resistant microbial pathogen in the foreseeable future.

In general, the distinct antibacterial activity of UKM8-ME could be due to phenol as the most abundant compound. Moreover, analytical data observations in this study showed compatibility with the literature. For example, Nowacka et al. (Nowacka et al., 2015) studied the antibacterial activity of phenolic substances 4-hydroxybenzoic acid, syringic acid, p-coumaric, and ferulic acids against wide range of Gram-positive and Gram-negative bacteria. This could be attributed to our findings owing to more potent antibacterial activity against Gram-positive bacteria (*S. epidermidis*, *S. aureus*, *B. subtilis*) with MICs values ranging from 0.313 to 5 mg/mL. Similarly, another study found greater antibacterial inhibition of phenols against the Gram-positive bacteria *S. aureus* (MIC 2.5 mg/mL) compared to the Gram-negative *E.coli* and *S. typhimurium* (MIC 5 mg/mL) (Zhao et al., 2021).

The antibacterial activity in phenolic compounds could be related to the capability of these substances to alter cell permeability (Alshuniaber et al., 2021). Also, these compound interact with proteins and enzymes in the microbial cell membrane, resulting in disruption of cellular function or genes. The disruption of cell cause degradation to amino acids responsible for microbial germination (El-baky et al., 2008).

Another theory of compounds responsible for the antimicrobial activity of UKM8-ME could be possibly linked with the synergistic action of phenol and the mixture matrices of compounds in the extract. It was considered that the antimicrobial activities of algae extracts could be closely related to a specific or with a mixture of compounds (Plaza et al., 2010, Pratt et al., 1944). Phenolic compounds combined with other substances such as fatty acids, terpenes or halogenated compounds were reported to be potentially new solutions to inhibit the microbes (Jimenez-Lopez et al., 2021, Shafay et al., 2016). However, a deeper analysis would be necessary to establish the antibacterial specificity or synergistic between identified compounds.

To the best of our knowledge, some identified compounds in this study include 8,11-Octadecadienoic acid, 1-bromodocosane and Cis-8-methyl-*exo*-tricyclo[5.2.1.0(2.6)]decane are the first reported in *Chlorella* in this study. In contrast, 7,10-Hexadecadienoic acid was identified in *Chlorella* (Cordeiro, 2020). However, this compound was not documented as an antimicrobial agent. Thus, the identified compounds could be considered as uniquely presented in species *Chlorella*.

## 5 Conclusion

This study reported new natural antimicrobial compounds found in the methanolic extract (ME) of three local microalgae isolates, *C. sorokiniana* UKM2, *Chlorella.* sp. UKM8, and *Scenedesmus* sp UKM9. Thus far this is the first report of antimicrobial activity of these three local isolates. UKM8-ME showed a profound antibacterial activity compared to the other two extracts against all selected bacteria with no cytotoxicity towards Vero cells. GC–MS analysis revealed fatty acids, alkanes, alkenes, phenol, and phytol, as the prominent antimicrobial compounds in UKM8-ME. More comprehensive studies are required to understand these antimicrobial compounds interactions and mechanism in UKM8- ME to unleash their specific potential.

## Declaration of Competing Interest

The authors declare that they have no known competing financial interests or personal relationships that could have appeared to influence the work reported in this paper.

## References

[b0005] Abdelrheem D.A., Rahman A.A., Elsayed K.N.M., Ahmed S.A. (2020). GC/MS spectroscopic approach, antimicrobial activity and cytotoxicity of some marine macroalgae from Qusier and Marsa Alam Seashore (Red Sea). Egypt. Egypt. J. Aquat. Biol. Fish..

[b0010] Adhoni S.A., Thimmappa S.C., Kaliwal B.B. (2016). Phytochemical analysis and antimicrobial activity of *Chorella vulgaris* isolated from Unkal Lake. J. Coast. Life Med..

[b0015] Alshuniaber M.A., Krishnamoorthy R., AlQhtani W.H. (2021). Saudi Journal of Biological Sciences Antimicrobial activity of polyphenolic compounds from *Spirulina* against food-borne bacterial pathogens. Saudi J. Biol. Sci..

[b0020] Bajpai V.K. (2016). Antimicrobial bioactive compounds from marine algae: A mini review. Indian J. Geo-Marine Sci..

[b0025] Balouiri M., Sadiki M., Ibnsouda S.K. (2016). Methods for in-vitro evaluating antimicrobial activity: A review. J. Pharm. Anal..

[b0030] Bauer A.W., Kirby W.M.M., Sherris J.C., Turck M. (1966). Antibiotic susceptibility testing by a standardized single disk method. Am. J. Clin. Pathol..

[b0035] Bhaigybati T., Gurumayum J., Ranjit Singh L., Grihanjali Devi P., Sanasam S., Bag G. (2020). Phytochemical profiling, antioxidant activity, antimicrobial activity and GC-MS analysis of *Ipomoea aquatica* Forsk collected from EMA market, Manipur.. J. Pharmacogn. Phytochem..

[b0040] Caldwell G.S. (2009). The influence of bioactive oxylipins from marine diatoms on invertebrate reproduction and development. Mar. Drugs.

[b0045] Chanda S., Rakholiya K. (2011). Combination therapy : Synergism between natural plant extracts and antibiotics against infectious diseases. Formatex.

[b0050] Cifuentes, A., Reglero, G., Herrero, M., Iba, E., Santoyo, S., 2006. Dunaliella salina Microalga Pressurized Liquid Extracts as Potential Antimicrobials 69, 2471–2477. 10.4315/0362-028x-69.10.2471 17066930

[b0055] Cordeiro, N., 2020. Hemiselmis andersenii and Chlorella stigmatophora as new sources of high-value compounds : a lipidomic approach 1 1–12. https://doi.org/10.1111/jpy.13042. 10.1111/jpy.13042 32683702

[b0060] Coronado-reyes J.A., Salaza-torres J.A., Juurez-campos B., Gonzalez-hernaned J.C. (2020). *Chlorella vulgaris*, a microalgae important to be used in Biotechnology: a review. Food Sci. Technol..

[b0065] de Felício R., de Albuquerque S., Young M.C.M., Yokoya N.S., Debonsi H.M. (2010). Trypanocidal, leishmanicidal and antifungal potential from marine red alga *Bostrychia tenella J. Agardh* (Rhodomelaceae, Ceramiales). J. Pharm. Biomed. Anal..

[b0070] de Morais M.G., Vaz B.d.S., de Morais E.G., Costa J.A.V. (2015). Biologically active metabolites synthesized by microalgae. Biomed Res. Int..

[b0075] Demirel Z., Yilmaz-Koz F., Karabay-Yavasoglu U., Ozdemir G., Sukatar A. (2009). Antimicrobial and antioxidant activity of brown algae from the *Aegean Sea*. J. Serbian Chem. Soc..

[b0080] Desbois A.P., Smith V.J. (2010). Antibacterial free fatty acids: Activities, mechanisms of action and biotechnological potential. Appl. Microbiol. Biotechnol..

[b0085] El-baky, H.H.A., Baz, F.K. El, Baroty, G.S.E., 2008. Evaluation of Marine Alga Ulva lactuca L . as A Source of Natural Preservative Ingredient 3, 434–444.

[b0090] Elshobary M.E., El-Shenody R.A., Ashour M., Zabed H.M., Qi X. (2020). Antimicrobial and antioxidant characterization of bioactive components from Chlorococcum minutum. Food Biosci..

[b0095] Farooqui M.N., Alrabie A., Basaꞌar O., Al-Qadsy I. (2019). Gc-Ms analysis, HPTLC fingerprint profile and DPPH free radical scavenging assay of methanol extract of Martynia annua linn seeds. Int. J. Pharm. Pharm. Sci..

[b0100] Fayyad A.G.S., Ibrahim N., Yaakob W.A. (2014). Phytochemical screening and antiviral activity of *Marrubium vulgare*. Malays. J. Microbiol..

[b0105] Flickinger, B.D., Huth, P.J., 2004. Dietary Fats and Oils : Technologies for Improving Cardiovascular Health. 10.1007/s11883-004-0088-4 15485593

[b0110] Gheda S.F., Ismail G.A. (2020). Natural products from some soil cyanobacterial extracts with potent antimicrobial, antioxidant and cytotoxic activities. An. Acad. Bras. Cienc..

[bib387] Thamilvanan D., Karthikeyan D., Muthukumaran M., Balakumar B.S (2016). Antibacterial activity of selected microalgal members of Chlorophyceae. World. J. Pharmacy Pharm. Sci..

[b0120] Guedes A.C., Gião M.S., Seabra R., Ferreira A.C.S., Tamagnini P., Moradas-Ferreira P., Malcata F.X. (2013). Evaluation of the antioxidant activity of cell extracts from microalgae. Mar. Drugs.

[b0125] Hariz H.B., Takriff M.S., Mohd Yasin N.H., Ba-Abbad M.M., Mohd Hakimi N.I.N. (2019). Potential of the microalgae-based integrated wastewater treatment and CO2 fixation system to treat Palm Oil Mill Effluent (POME) by indigenous microalgae; *Scenedesmus* sp. and *Chlorella* sp. J. Water Process Eng..

[b0130] Hassan M.H.A., Mohammed R., Hetta M.H., Abdelaziz T.A., El-Gendy A.O., Sleim M.A. (2016). Biological and chemical investigation of the soft coral *lobophytum pauciflorum* collected from the Egyptian Red Sea. Int. J. Pharmacogn. Phytochem. Res..

[b0135] Hoek, C., Mann, D., Jahns, H.M., Jahns, M., 1995. Algae: an introduction to phycology.

[bib390] Hazman N.A.S., Mohd Yasin N.H., Takriff M.S., Hasan H.A., Kamarudin K.F., Hakimi N.I.N.M (2018). Integrated palm oil mill effluent and CO_2_ sequestration by microalgae. Sains Malaysiana.

[b0140] Hussein H.A., Syamsumir D.F., Radzi S.A.M., Siong J.Y.F., Zin N.A.M., Abdullah M.A. (2020). Phytochemical screening, metabolite profiling and enhanced antimicrobial activities of microalgal crude extracts in co-application with silver nanoparticle. Bioresour. Bioprocess..

[b0145] Islam M.T., Ali E.S., Uddin S.J., Shaw S., Islam M.A., Ahmed M.I., Chandra Shill M., Karmakar U.K., Yarla N.S., Khan I.N., Billah M.M., Pieczynska M.D., Zengin G., Malainer C., Nicoletti F., Gulei D., Berindan-Neagoe I., Apostolov A., Banach M., Yeung A.W.K., El-Demerdash A., Xiao J., Dey P., Yele S., Jóźwik A., Strzałkowska N., Marchewka J., Rengasamy K.R.R., Horbańczuk J., Kamal M.A., Mubarak M.S., Mishra S.K., Shilpi J.A., Atanasov A.G. (2018). Phytol: A review of biomedical activities. Food Chem. Toxicol..

[b0150] Jafari, S., Mobasher, M.A., Najafipour, S., Ghasemi, Y., Mohkam, M., Ebrahimi, M.A., Mobasher, N., 2018. Antibacterial potential of Chlorella vulgaris and Dunaliella salina extracts against Streptococcus mutans. Jundishapur J. Nat. Pharm. Prod. https://doi.org/10.5812/jjnpp.13226

[b0345] Japar A.S., Takriff M.S., Mohd Yasin N.H. (2021). Microalgae acclimatization in industrial wastewater and its effect on growth and primary metabolite composition. Algal Res..

[b0155] Jimenez-Lopez C., Pereira A.G., Lourenço-Lopes C., Garcia-Oliveira P., Cassani L., Fraga-Corral M., Prieto M.A., Simal-Gandara J. (2021). Main bioactive phenolic compounds in marine algae and their mechanisms of action supporting potential health benefits. Food Chem..

[b0160] Jodallah N., saleem Ali-shtayeh M. (2013).

[b0165] Kamat S., Kumari M., Taritla S., Jayabaskaran C. (2020). Endophytic fungi of marine a lga From Konkan Coast, India—A rich Source of bioactive material. Front. Mar. Sci..

[b0170] Kamei Y., Isnansetyo A. (2003). Lysis of methicillin-resistant *Staphylococcus aureus* by 2,4-diacetylphloroglucinol produced by *Pseudomonas* sp. AMSN isolated from a marine alga. Int. J. Antimicrob. Agents.

[b0175] Karabay-Yavasoglu N.U., Sukatar Atakan, Ozdemir Guven, Horzum Zerrin (2007). Antimicrobial activity of volatile components and various extracts of the red alga Jania rubens. Phytother. Res..

[b0180] Kellam S.J., Walker J.M. (1989). Antibacterial activity from marine microalgae in laboratory culture. Br. Phycol. J..

[b0185] Kumar Prasun, Lee Jin-Hyung, Beyenal Haluk, Lee Jintae (2020). Fatty acids as antibiofilm and antivirulence agents. Trends Microbiol..

[b0190] Landsberg J.H. (2002). The effects of harmful algal blooms on aquatic organisms. Rev. Fish. Sci..

[b0195] Lauritano C., Andersen J.H., Hansen E., Albrigtsen M., Escalera L., Esposito F., Helland K., Hanssen K.Ø., Romano G., Ianora A. (2016). Bioactivity screening of microalgae for antioxidant, anti-inflammatory, anticancer, anti-diabetes, and antibacterial activities. Front. Mar. Sci..

[b0200] Lv J.S., Zhang L.N., Song Y.Z., Wang X.F., Chu X.Z. (2011). Biological activity exhibited by secondary metabolites of the Albizia julibrissin Durazz. pod. Int. Biodeterior. Biodegrad..

[b0205] Maadane A., Merghoub N., Ainane T., El Arroussi H., Benhima R., Amzazi S., Bakri Y., Wahby I. (2015). Antioxidant activity of some Moroccan marine microalgae: Pufa profiles, carotenoids and phenolic content. J. Biotechnol..

[b0210] Makridis P, Costa R.A., Dinis M.T. (2006). Microbial conditions and antimicrobial activity in cultures of two microalgae species, *Tetraselmis chuii* and *Chlorella minutissima*, and effect on bacterial load of enriched *Artemia metanauplii*. Aquaculture.

[b0215] Malebo, H.M., Tanja, W., Cal, M., Swaleh, S.A.M., Omolo, M.O., Hassanali, A., 2009. activity of selected Tanzanian medicinal plants 11, 226–234. 10.4314/thrb.v11i4.50194 20734703

[b0220] Marrez D.A., Naguib M.M., Sultan Y.Y., Higazy A.M. (2019). Antimicrobial and anticancer activities of Scenedesmus obliquus metabolites. Heliyon.

[b0225] Mimouni V., Ulmann L., Pasquet V., Mathieu M., Picot L., Bougaran G., Cadoret J.-P., Morant-Manceau A., Schoefs B. (2012). The potential of microalgae for the production of bioactive molecules of pharmaceutical interest. Curr. Pharm. Biotechnol..

[b0230] Mohamed S.S., Saber A.A. (2019). Antifungal potential of the bioactive constituents in extracts of the mostly untapped brown seaweed *Hormophysa cuneiformis* from the Egyptian coastal waters. Egypt. J. Bot..

[b0235] Mudimu O., Friedl T., Rybalka N., Bauersachs T., Schulz R., Born J. (2014). Biotechnological screening of microalgal and cyanobacterial strains for biogas production and antibacterial and antifungal effects. Metabolites.

[b0240] Nand P., Drabu S., Gupta R.K. (2011). Antimicrobial investigation of *Linum usitatissimum* for the treatment of acne. Nat. Prod. Commun..

[b0245] Nowacka, N., Nowak, R., Drozd, M., Olech, M., Los, R., 2015. Antibacterial , Antiradical Potential and Phenolic Compounds of Thirty-One Polish Mushrooms 1–13. https://doi.org/10.1371/journal.pone.0140355. 10.1371/journal.pone.0140355 PMC4607371 26468946

[b0250] Olasehinde T.A., Odjadjare E.C., Mabinya L.V., Olaniran A.O., Okoh A.I. (2019). *Chlorella sorokiniana* and *Chlorella minutissima* exhibit antioxidant potentials, inhibit cholinesterases and modulate disaggregation of β-amyloid fibrils. Electron. J. Biotechnol..

[b0255] Owen Lucy, White Alex W., Laird Katie (2019). Characterisation and screening of antimicrobial essential oil components against clinically important antibiotic-resistant bacteria using thin layer chromatography-direct bioautography hyphenated with GC-MS, LC-MS and NMR. Phytochem. Anal..

[b0260] Ozdemir, G., Karabay, N.U., Dalay, M.C., Pazarbasi, B., 2004. Antibacterial activity of volatile component and various extracts of Spirulina platensis 757, 754–757. 10.1002/ptr.1541 15478198

[b0265] Patil Lakkanagouda, Kaliwal B.B. (2019). Microalga *Scenedesmus bajacalifornicus* BBKLP-07, a new source of bioactive compounds with in vitro pharmacological applications. Bioprocess Biosyst. Eng..

[b0270] Patra J.K., Patra A.P., Mahapatra N.K., Thatoi H.N., Das S., Sahu R.K., Swain G.C. (2009). Antimicrobial activity of organic solvent extracts of three marine macroalgae from Chilika Lake, Orissa. India. Malays. J. Microbiol..

[b0275] Phang, S., Mustafa, E.M., Ambati, R.R., Meriam, N., Sulaiman, N., Lim, P., Majid, N.A., Dommange, X., 2015. Checklist of microalgae collected from different habitats in peninsular malaysia for selection of algal biofuel feed-stocks 34.

[b0280] Plaza Merichel, Santoyo Susana, Jaime Laura, Avalo Belkis, Cifuentes Alejandro, Reglero Guillermo, García-Blairsy Reina Guillermo, Señoráns Francisco Javier, Ibáñez Elena (2012). Comprehensive characterization of the functional activities of pressurized liquid and ultrasound-assisted extracts from *Chlorella vulgaris*. LWT - Food Sci. Technol..

[b0285] Plaza M., Santoyo S., Jaime L., García-Blairsy Reina G., Herrero M., Señoráns F.J., Ibáñez E. (2010). Screening for bioactive compounds from algae. J. Pharm. Biomed. Anal..

[b0290] Prestinaci, F., Pezzotti, P., Pantosti, A., 2015. Antimicrobial resistance: A global multifaceted phenomenon. Pathog. Glob. Health. https://doi.org/10.1179/2047773215Y.0000000030 10.1179/2047773215Y.0000000030 PMC4768623 26343252

[b0295] Ramasamy V. (2014). Chemical composition of *Spirulina* by gas chromatography coupled with mass spectrophotometer (GC-MS). Int. J. Pharm. Phytopharm. Res..

[b0300] Reichelt John L., Borowitzka Michael A. (1984). Antimicrobial activity from marine algae: Results of a large-scale screening programme. Hydrobiologia.

[b0305] Robertson Pratt T.C., Daniels J.J., Eiler J.B., Gunnison W.D., Kumler J.F., Oneto L.A., Strait H.A., Spoehr G.J., Hardin H.W., Milner J.H.C., Smith H.H.S. (1944). Chlorellin, an antibacterial sub- stance from *Chlorella*. Science (80-.).

[b0310] Saritha, S., Kala, K.J., J, P.P.K., Nair, S.M., 2017. In vitro antibacterial screening of fatty acid fractions from three different microalgae 9, 1405–1409. https://doi.org/10.25258/phyto.v9i11.11182.

[bib388] Salem O.M., Hoballah E.M., Ghazi S.M., Hanna S.N. (2014). Antimicrobial activity of microalgal extracts with special emphasize onNostoc sp.. Life. Sci. J..

[bib386] Santhosh S., Manivannan N., Ragavendran C., Mathivanan N., Natarajan D., Hemalatha N., Dhandapani R. (2019). Growth optimization, free radical scavenging and antibacterial potential of Chlorella sp. SRD3 extracts against clinical isolates. J. Appl. Microbiol..

[b0315] Sawant S.S., Mane V.K. (2018). Nutritional profile, antioxidant, antimicrobial potential, and bioactives profile of *Chlorella emersonii* kj725233. Asian J. Pharm. Clin. Res..

[bib389] Ding G.T., Mohd Yasin N.H., Takriff M.S., Kamarudin K.F., Salihon J., Yaakob Z., Hakimi N.I.N.M. (2020). Phycoremediation of palm oil mill effluent (POME) and CO2 fixation by locally isolated microalgae: Chlorella sorokiniana UKM2, Coelastralla sp. UKM4 and Chlorella pyrenoidosa UKM7. J. Water. Proc. Eng..

[b0320] El Shafay S.M., Ali S.S., El-Sheekh M.M. (2016). Antimicrobial activity of some seaweeds species from Red sea, against multidrug resistant bacteria. Egypt. J. Aquat. Res..

[b0325] Shaima A.F., Gires U., Asmat A. (2016). Algicidal activity of Aeromonas hydrophila sdPS-7 isolate against toxic marine dinoflagellate Alexandrium minutum KB-5. Malays. J. Microbiol..

[b0330] Shannon E., Abu-Ghannam N. (2016). Antibacterial derivatives of marine algae: An overview of pharmacological mechanisms and applications. Mar. Drugs..

[b0335] Silva A., Silva S.A., Lourenço-Lopes C., Jimenez-Lopez C., Carpena M., Gullón P., Fraga-Corral M., Domingues V.F., Fátima Barroso M., Simal-Gandara J., Prieto M.A. (2020). Antibacterial use of macroalgae compounds against foodborne pathogens. Antibiotics.

[b0340] Sit N.W., Chan Y.S., Lai S.C., Lim L.N., Looi G.T., Tay P.L., Tee Y.T., Woon Y.Y., Khoo K.S., Ong H.C. (2018). In vitro antidermatophytic activity and cytotoxicity of extracts derived from medicinal plants and marine algae. J. Mycol. Med..

[b0350] Van Meerloo J., Kaspers G.J.L., Cloos J. (2011). Cell sensitivity assays: The MTT assay. Methods Mol. Biol..

[b0355] Vikneshan M., Saravanakumar R., Mangaiyarkarasi R., Rajeshkumar S., Samuel S.R., Suganya M., Baskar G. (2020). Saudi Journal of Biological Sciences Algal biomass as a source for novel oral nano-antimicrobial agent. Saudi J. Biol. Sci..

[b0360] Kumar Vinay (2011). Antibacterial activity of crude extracts of *Spirulina platensis* and its structural elucidation of bioactive compound. J. Med. Plants Res..

[b0365] Wolfe G.V., Strom S.L., Holmes Jan L., Radzio T., Olson M.B. (2002). Dimethylsulfoniopropionate cleavage by marine phytoplankton in response to mechanical, chemical, or dark stress1. J. Phycol..

[b0370] Yap W.-F., Tay V., Tan S.-H. (2019). Decoding coding antioxidant and antibacterial potentials of Malaysian green seaweeds: Caulerpa racemosa and Caulerpa lentillifera. Antibiotics.

[b0375] Zea-Obando C., Tunin-Ley A., Turquet J., Culioli G., Briand J.F., Bazire A., Réhel K., Faÿ F., Linossier I. (2018). Anti-bacterial adhesion activity of tropical microalgae extracts. Molecules.

[b0380] Zhao Meimei, Bai Jingwen, Bu Xueying, Tang Yang, Han Wenqing, Li Dalong, Wang Libo, Yang Y., Xu Yaqin (2021). Microwave-assisted aqueous two-phase extraction of phenolic compounds from Ribes nigrum L. and its antibacterial effect on foodborne pathogens. Food Control.

[b0385] Zielinski Dariusz, Fraczyk Justyna, Debowski Marcin, Zielinski Marcin, Kaminski Zbigniew J., Kregiel Dorota, Jacob Claus, Kolesinska Beata (2020). Biological activity of hydrophilic extract of *Chlorella vulgaris* grown on post-fermentation leachate from a biogas plant supplied with stillage and maize silage. Molecules.

